# Chloroplast Redox Status Modulates Genome-Wide Plant Responses during the Non-host Interaction of Tobacco with the Hemibiotrophic Bacterium *Xanthomonas campestris* pv. *vesicatoria*

**DOI:** 10.3389/fpls.2017.01158

**Published:** 2017-07-04

**Authors:** Juan J. Pierella Karlusich, Matias D. Zurbriggen, Fahimeh Shahinnia, Sophia Sonnewald, Uwe Sonnewald, Seyed A. Hosseini, Mohammad-Reza Hajirezaei, Néstor Carrillo

**Affiliations:** ^1^Instituto de Biología Molecular y Celular de Rosario (Consejo Nacional de Investigaciones Científicas y Técnicas), Facultad de Ciencias Bioquímicas y Farmacéuticas, Universidad Nacional de RosarioRosario, Argentina; ^2^Leibniz Institute of Plant Genetics and Crop Plant ResearchGatersleben, Germany; ^3^Department of Biology, Division of Biochemistry, Friedrich-Alexander-University Erlangen-NurembergErlangen, Germany

**Keywords:** plant-microbe interactions, chloroplast redox status, reactive oxygen species, transcriptomics, flavodoxin, localized cell death

## Abstract

Non-host resistance is the most ample and durable form of plant resistance against pathogen infection. It includes induction of defense-associated genes, massive metabolic reprogramming, and in many instances, a form of localized cell death (LCD) at the site of infection, purportedly designed to limit the spread of biotrophic and hemibiotrophic microorganisms. Reactive oxygen species (ROS) have been proposed to act as signals for LCD orchestration. They are produced in various cellular compartments including chloroplasts, mitochondria and apoplast. We have previously reported that down-regulation of ROS build-up in chloroplasts by expression of a plastid-targeted flavodoxin (Fld) suppressed LCD in tobacco leaves inoculated with the non-host bacterium *Xanthomonas campestris* pv. *vesicatoria* (*Xcv*), while other defensive responses were unaffected, suggesting that chloroplast ROS and/or redox status play a major role in the progress of LCD. To better understand these effects, we compare here the transcriptomic alterations caused by *Xcv* inoculation on leaves of Fld-expressing tobacco plants and their wild-type siblings. About 29% of leaf-expressed genes were affected by *Xcv* and/or Fld. Surprisingly, 5.8% of them (1,111 genes) were regulated by Fld in the absence of infection, presumably representing pathways responsive to chloroplast ROS production and/or redox status during normal growth conditions. While the majority (∼75%) of pathogen-responsive genes were not affected by Fld, many *Xcv* responses were exacerbated, attenuated, or regulated in opposite direction by expression of this protein. Particularly interesting was a group of 384 genes displaying *Xcv* responses that were already triggered by Fld in the absence of infection, suggesting that the transgenic plants had a larger and more diversified suite of constitutive defenses against the attacking microorganism compared to the wild type. Fld modulated many genes involved in pathogenesis, signal transduction, transcriptional regulation and hormone-based pathways. Remarkable interactions with proteasomal protein degradation were observed. The results provide the first genome-wide, comprehensive picture illustrating the relevance of chloroplast redox status in biotic stress responses.

## Introduction

Plant pathogens are classified as necrotrophs, biotrophs, or hemibiotrophs based on their modes of nutrition and infection strategies (Mengiste, 2012; Spanu, 2012; Fatima and Senthil-Kumar, 2015). Necrotrophs kill host cells and feed on them, whereas biotrophs require living tissue to grow, and consequently manipulate host physiology to obtain nutrients. Hemibiotrophs usually display a biotrophic stage of nutrition before shifting to a necrotrophic lifestyle (Spanu, 2012; Fatima and Senthil-Kumar, 2015).

Plants, in turn, deploy a multi-layered response to oppose pathogen invasion, with both constitutive and inducible elements. A first line of defense is made up of preformed obstacles that restrict access of the microorganism to plant cells, such as the cuticle layer and cell wall, as well as constitutively produced compounds with antimicrobial activity (Senthil-Kumar and Mysore, 2013). If these defenses are overcome, induced responses provide the main contribution to plant resistance. They are initiated by perception of evolutionary conserved pathogen-associated molecular patterns (PAMPs), such as components of the flagellum or the lipopolysaccharide, by plant extracellular receptors (Dangl et al., 2013; Gill et al., 2015). The resulting defensive responses, collectively known as PAMP-triggered immunity (PTI), attempt to arrest pathogen growth by a number of mechanisms including cell wall reinforcement, synthesis of antimicrobials and increased expression of pathogenesis-related (PR) proteins (Senthil-Kumar and Mysore, 2013). In the course of evolution, some microorganisms acquired the ability to transfer effector proteins into the plant cell which target PAMP-induced responses and render them ineffectual. The plant then becomes a susceptible host for the effector-carrying pathogen, eventually leading to disease. Under selection pressure, a host plant may undergo changes in the surveillance machinery, resulting in new variants that are able to recognize and neutralize the effectors. These plants gain resistance based on the action of one or a few genes (termed *R* genes), and the next level of defensive responses is initiated, referred to as effector-triggered immunity or ETI (Dangl et al., 2013; Cui et al., 2015). ETI is generally a stronger deterrent for pathogen spread than PTI, and often involves a hypersensitive reaction (HR), a multigenic process that leads in most cases to localized cell death (LCD) at the site of infection (Senthil-Kumar and Mysore, 2013).

Depending on their nature, the interactions established between plant and microorganism can be classified as host or non-host (Mysore and Ryu, 2004). They are usually distinguished by the adaptation of the microorganism to a particular plant cultivar (host) and a lack of adaptation to others (non-host). Host resistance is largely cultivar-specific and relies on individual *R* genes present in the plant. In contrast, non-host resistance is displayed by all cultivars of a plant species against all races of a particular microorganism and is more durable (Mysore and Ryu, 2004; Senthil-Kumar and Mysore, 2013; Gill et al., 2015). It involves both PTI and ETI and is thus often accompanied by HR and LCD symptoms (Senthil-Kumar and Mysore, 2013). It is assumed that the LCD associated to the HR helps to contain biotrophic or hemibiotrophic pathogens by opposing a barrier of dead cells which deter their advance into the adjacent living tissue (Senthil-Kumar and Mysore, 2013).

Localized cell death is a genetically controlled process involving changes in the expression of many genes (Kurusu et al., 2015). Increased generation of reactive oxygen species (ROS), such as singlet oxygen, superoxide and hydrogen peroxide, commonly precedes tissue death, and the role of oxidants in triggering and/or executing LCD is supported by several lines of evidence (Montillet et al., 2005; Torres and Dangl, 2005; Delprato et al., 2015). ROS can be produced in various cellular compartments: in the apoplast by dedicated oxidases bound to the plasma membrane, and intracellularly in chloroplasts, mitochondria and peroxisomes, as byproducts of metabolic processes such as photosynthesis, respiration and photorespiration (Mittler et al., 2004; Torres et al., 2006; Mühlenbock et al., 2008; Mur et al., 2008). The relative contribution of ROS synthesized in different compartments to leaf LCD has yet to be established. By comparison with mammalian systems, extracellular ROS generation was regarded as more important for the establishment of LCD (Morales et al., 2016), although chloroplasts and peroxisomes are the main source of ROS in the light (Foyer and Noctor, 2003; Mur et al., 2008). Indeed, several reports have shown that full manifestation of leaf LCD during plant-pathogen interactions requires light, and is delayed or abolished in the dark (Liu et al., 2007; Kim et al., 2012; Rodríguez Hervá et al., 2012; Delprato et al., 2015).

Reactive oxygen species accumulation in chloroplasts during stress episodes can be selectively controlled by the expression of a plastid-targeted cyanobacterial flavodoxin (Fld). Flds are flavin mononucleotide-containing electron carrier proteins whose expression is induced in phototrophic microorganisms in response to environmental hardships and iron starvation (Sancho, 2006; Pierella Karlusich et al., 2014). Under these adverse conditions, the photosynthetic electron transport chain (PETC) becomes over-reduced due to limitation of terminal electron acceptors (ferredoxin and NADP^+^), and the excess of excitation energy and reducing equivalents can be misrouted to oxygen, thus increasing the rates of ROS formation (Pierella Karlusich et al., 2014; Pierella Karlusich and Carrillo, 2017). When present, Fld provides an alternative electron sink which drives reducing equivalents away from oxygen and into productive pathways, effectively preventing over-reduction of the PETC (Zurbriggen et al., 2008; Pierella Karlusich et al., 2014). Then, Fld affects the redox poise of the PETC besides decreasing plastid ROS production. While this flavoprotein is not found in plants (Pierella Karlusich et al., 2015), introduction of a plastid-targeted Fld in various plant species resulted in lower ROS accumulation and increased tolerance to multiple sources of stress, including drought, extreme temperatures, excess irradiation and iron deficit (Tognetti et al., 2006, 2007; Zurbriggen et al., 2008; Coba de la Peña et al., 2010; Li et al., 2016; Lodeyro et al., 2016). The results indicated that the cyanobacterial Fld was able to productively interact with the plant PETC and behave as a general antioxidant specific for chloroplasts.

We used these transgenic lines as a tool to probe the role of chloroplast redox status (including ROS propagation) in the execution of LCD during biotic interactions. Tobacco plants expressing Fld accumulated less ROS in the chloroplasts of leaves infiltrated with the hemibiotrophic bacterium *Xanthomonas campestris* pv. *vesicatoria* (*Xcv*). This microorganism is the causal agent of bacterial spot disease in pepper and tomato (Schulte and Bonas, 1992). It was chosen to study the involvement of plastidic ROS in biotic interactions because *Xcv* elicits a strong HR with LCD without causing disease in any described tobacco cultivar, thus conforming to a typical non-host interaction (Adlung et al., 2016), and allowing comparison of the pathogen-induced responses of Fld-expressing and non-expressing plants in a manner that is independent of disease development (Zurbriggen et al., 2009). Down-regulation of chloroplast ROS levels in Fld-expressing plants correlated with the suppression of *Xcv*-dependent LCD symptoms in the inoculated tissue (Zurbriggen et al., 2009), indicating that the chloroplast redox status and/or ROS build-up play important role(s) in triggering LCD during this non-host interaction. Noteworthy, other events associated with the HR, such as the induction of PR genes and the increase of salicylic acid (SA) and jasmonic acid (JA) levels, proceeded as in the wild type (Zurbriggen et al., 2009).

Non-host resistance, HR and LCD involve massive changes of gene expression patterns in the affected tissue (Daurelio et al., 2011). The objective of this article is to determine how extensively the genetic reprogramming induced by interaction with the microorganism is influenced by Fld expression and decrease of chloroplast ROS, with the aim of characterizing at the molecular level the relationships between chloroplast redox status and ROS formation on one side, and host responses to pathogen attack on the other. We used microarray hybridization techniques to generate genome-wide transcript profiles from WT and Fld-expressing tobacco leaves inoculated with *Xcv* or a mock solution. Analysis of transcriptomic data revealed that a significant fraction (∼25%) of the thousands of genes differentially expressed (DE) in response to *Xcv* were affected by the presence of chloroplast Fld, including genes encoding PR proteins and components of central metabolic pathways, signal transduction and transcriptional regulation. It also allowed the identification of numerous genes whose expression was altered by Fld in the absence of *Xcv* infiltration. The results provide a detailed picture of how chloroplast ROS and/or redox poise affect genetic and metabolic reprogramming during this type of plant-microbe interaction.

## Materials and Methods

### Plant Material and *Xcv* Inoculation

The design and preparation of homozygous *pfld* and *cfld* lines of tobacco (*Nicotiana tabacum* cv. Petit Havana), expressing Fld in plastids and cytosol, respectively, have been described elsewhere (Tognetti et al., 2006; Ceccoli et al., 2012). Independent lines *pfld*4-2 and *pfld*5-8 accumulated similar levels of Fld (60–70 pmol Fld g^-1^ fresh weight, in the same order of endogenous Fd), and displayed equivalent protection against biotic and abiotic stresses (Tognetti et al., 2006, 2007; Zurbriggen et al., 2009).

Plants were grown in soil at a light intensity of 250 μmol quanta m^-2^ s^-1^, with a 16-h photoperiod and a relative humidity of 80% (greenhouse conditions). The *Xcv* Bv5-4a strain (Doidge) from the stock collection of the National Institute of Agricultural Technology (INTA Bella Vista, Argentina) was used for the inoculation experiments. *Xcv* cells were cultured at 28°C in a modified yeast extract–peptone–dextrose medium containing 1% (w/v) dextrose, 1% (w/v) bactopeptone, 1% (w/v) yeast extract, pH 7.3. An overnight culture was diluted (1:100) in fresh broth and grown for additional 18 h. Cells were collected by centrifugation (15 min at 700 *g*), washed with 10 mM MgCl_2_ and finally resuspended in the same solution. Bacterial suspensions corresponding to 10^8^ colony forming units (CFU) mL^-1^ were inoculated on the abaxial side of one half of the second youngest fully expanded leaves of 6-week-old WT and transgenic tobacco plants using a needle-less plastic syringe, whereas the other half of the same leaf was infiltrated with 10 mM MgCl_2_ as a mock control. After inoculation, plants were incubated at 250 μmol quanta m^-2^ s^-1^ until sampling.

### RNA Isolation, cDNA Labeling and Microarray Hybridization

For microarray analysis, two independent experiments were performed with *pfld*4-2 and WT plants from the same seed batch. Leaf material from *Xcv*- and mock-infiltrated tissue from 10 plants of each genotype and treatment was collected from each experiment at 19 h post-infiltration (hpi), frozen in liquid nitrogen and ground with Mixer Mill MM 400 (Retsch). Two pools of biological samples, each from two independent experiments per genotype and condition were used for the microarray analysis. Leaf RNA was extracted as described by Logemann et al. (1987). RNA quantity and quality were determined with a NanoDrop spectrophotometer (Thermo Scientific, Wilmington, DE, United States) and by visual inspection after electrophoresis, respectively. One microgram of RNA from each sample was treated with RQ1 DNase (Promega, Madison, WI, United States) according to the manufacturer’s instructions, and used as template to generate cDNA with M-MLV Reverse Transcriptase (Promega, Madison, WI, United States).

Gene expression profiles of tobacco leaves were assessed with a 60-mer oligobased 4 × 44k Agilent microarray (Tobacco Gene Expression Microarray design ID: 021113), consisting of 43,759 60-mer probes corresponding to 26,942 unigenes, which was designed mainly based on the Institute for Genomic Research (TIGR) and Unigenes in the NCBI databases. Sample labeling and hybridization were performed as described in the one-color microarray-based gene expression analysis protocol including the one-color RNA spike-in Kit according to the manufacturer’s instructions (v5.0.1, Agilent Technologies). Slides were scanned with an Agilent microarray scanner (G2505B) at high resolution. Data were extracted using feature extraction software (v9.5.3, Agilent Technologies) using a standard protocol.

### Microarray Data Analysis

Data processing and statistical analysis were carried out with the Bioconductor library limma (Ritchie et al., 2015). Background correction and normalization were performed using the ”normexp” and quantile methods, respectively. In the case of multiple probes corresponding to the same unigene, values were averaged. Probe-to-unigene assignments were carried out using the corresponding file (microarray_nta_Agilent_4x44k_genes.txt) available at the GoMapMan website resource^1^ (Ramšak et al., 2014). An empirical Bayes method with a moderated *t*-statistic was employed for the determination of the genes with statistically significant changes, whereas the Benjamini and Hochberg’s method was used to control false discovery rates (FDR). DE genes were identified from pairwise comparisons when FDR < 0.05 and fold-change (FC) > 2 or < 0.5.

Based on the results of the multiple comparison test described above, genes were defined as induced (FC > 2 and FDR < 0.05), repressed (FC < 0.5 and FDR < 0.05) or unaffected in each of the four pairwise comparison combinations (*pfld*4-2 vs. WT under mock conditions, *pfld*4-2 vs. WT under *Xcv* infiltration, mock vs. *Xcv* in *pfld*4-2 line, mock vs. *Xcv* in WT line). An *ad hoc* made R script was used to group the genes sharing the same results in the four pairwise comparisons. Graphics representing the resulting clusters were prepared with the R library ggplot2 (Wickham, 2009). Mapman ontology was used for probe annotation and functional assignment (Thimm et al., 2004) using a mapping file updated in September 2015 (nta_ntaUG17_2015-09-08_mapping.txt) from the GoMapMan website resource^1^ (Ramšak et al., 2014). The Mapman ontology consists of 35 major groups (called “BINs”) which are in turn subdivided into hierarchical structures. Genes can be assigned even when their function is approximate, and the same gene can belong to more than one BIN or subBIN (Thimm et al., 2004).

Pathway over-representation analyses between lines or treatment comparisons were performed with PageMan (Usadel et al., 2006). The analysis was carried out separately for induced (FC > 2 and FDR < 0.05) and repressed (FC < 0.5 and FDR < 0.05) genes using Fisher’s exact test with Bonferroni correction (FDR < 0.05). Pathway over-representation analysis in each cluster was also performed with PageMan (Usadel et al., 2006) using Fisher’s exact test with Bonferroni correction (FDR < 0.05).

### Validation of DE Genes by Quantitative Reverse-Transcription (qRT)-PCR

For qRT-PCR determinations, the analyzed samples corresponded to lines *pfld*4-2, *pfld*5-8, *pfld*5-4 (obtained by crossing *pfld*5-8 and WT plants, and therefore containing 50% Fld contents; Ceccoli et al., 2012), and *cfld*1-4 (which expresses high levels of Fld in the cytosol; Tognetti et al., 2006; Ceccoli et al., 2012). Total RNA was extracted from *Xcv*- and mock-infiltrated samples at 19 hpi, treated with DNAse I (Thermo Scientific) and reverse-transcribed with the RevertAid First Strand cDNA Synthesis kit (Thermo Scientific) using Oligo(dT)_18_ primer, according to the manufacturer’s instructions. To validate DE genes identified in the microarray, quantitative reverse-transcription (qRT)-PCR was carried out with IQ SYBR Green Supermix (BIO-RAD) in Eppendorf Realplex Mastercycle ep Gradient S using the following conditions: 95°C for 5 min and then 40 cycles of 95°C for 15 s, 60°C for 15 s, and 72°C for 20 s. Between 5 and 6 biological replicates were assayed for each reaction. The specificity of qRT-PCR products was confirmed by performing a melting temperature analysis and agarose gel electrophoresis detection followed by sequencing. The relative abundance of transcripts of the genes of interest was quantified with the delta threshold cycle (ΔΔ*C*_t_) method (Schmittgen and Livak, 2008), and normalized to the gene encoding the 60S ribosomal protein L25. Primers used in this study are listed in **Supplementary Table S1**. Each qRT-PCR reaction set included 5–6 biological and 2 technical replicates of samples, and water used as a negative no-template control instead of cDNA. Successful removal of DNA contamination was confirmed by the absence of PCR amplification products using samples of total RNA and specific primers of the Ribosomal L25 -encoding gene. The *L25r* transcript was used as the *housekeeping gene* and a control to normalize expression levels. The statistical analysis of the qPCR data was carried out by ANOVA using R language^2^.

### Availability of Supporting Data

Data reported in this publication have been deposited in NCBI’s Gene Expression Omnibus (Edgar et al., 2002) and are accessible through GEO Series accession number GSE92596^3^.

## Results and Discussion

### Microarray Analysis of WT and Fld-Expressing Plants Inoculated with *Xcv*

We have previously shown that Fld expression in tobacco chloroplasts did prevent plastid ROS build-up when leaves were challenged with the non-host microorganism *Xcv* (Zurbriggen et al., 2009, 2010). At 19 hpi with 10^8^ CFU mL^-1^ *Xcv*, ROS accumulated to high levels in the chloroplasts of WT leaves, but not in those expressing plastid-targeted Fld (see **Figure 1** in Zurbriggen et al., 2009). *Xcv*-treated WT leaves showed initial symptoms of yellowing and loss of turgor at this stage, which developed into full-blown LCD by 24 hpi (see **Figure 2** in Zurbriggen et al., 2009). Tissue death was almost entirely prevented in infiltrated *pfld*4-2 leaves. To further characterize the effect of chloroplast Fld expression during the tobacco-*Xcv* interaction, a genome-wide transcriptional profiling was carried out using WT and *pfld*4-2 leaves as a source of RNA. The conditions of Zurbriggen et al. (2009) were used to allow comparisons with the phenotypic results, and the sampling time was chosen at 19 hpi because differences in ROS levels between WT and *pfld* plants were maximal at this stage, whereas longer exposure to the microorganism led to rapid cellular collapse (Zurbriggen et al., 2009), and ROS ceased to be produced. Therefore, leaf tissue was infiltrated with 10^8^ CFU mL^-1^ or mock solution as described in Materials and Methods, and samples were collected at 19 hpi. Since *pfld*4-2 and *pfld*5-8 plants displayed essentially the same responses to *Xcv* infiltration (Zurbriggen et al., 2009), we performed the microarray analysis on *pfld*4-2 leaves, although the similar nature of *pfld*5-8 responses was confirmed during validation of the microarray data (see below).

**FIGURE 1:**
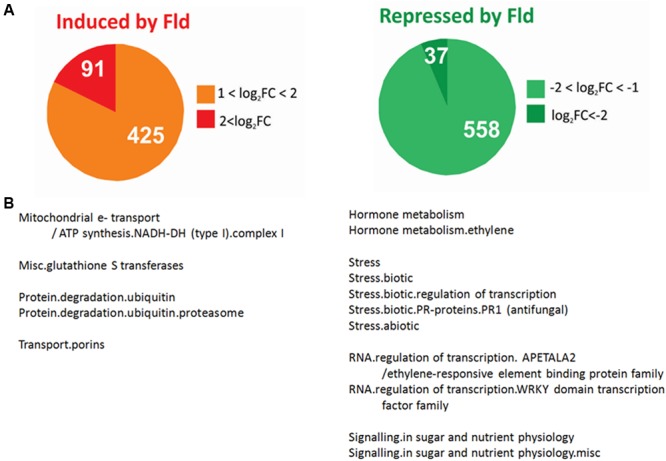
Effect of Fld expression on the tobacco transcriptome. **(A)** Pie charts showing the number of genes differentially induced (FC > 2 and FDR < 0.05) or repressed (FC < 0.5 and FDR < 0.05) by Fld in plants grown under greenhouse conditions. **(B)** Over-representation analysis of genes differentially expressed (DE) in response to Fld in Mapman functional pathways. The analysis was carried out separately for induced and repressed genes (Fisher’s exact test with Bonferroni correction with FDR < 0.05). The list of unigenes and their corresponding descriptions, pathway assignments and fold-change values are described in **Supplementary Table S2**. FC, fold-change.

**FIGURE 2:**
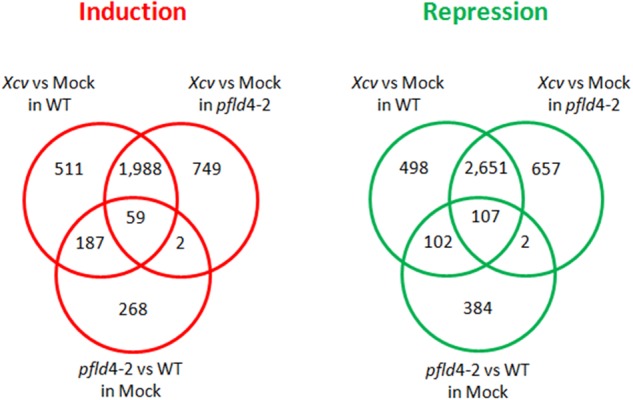
Venn diagrams of DE genes due to the effect of *Xcv* infection and Fld expression. Induced genes were defined when FC > 2 and FDR < 0.05, and repressed genes when FC < 0.5 and FDR < 0.05.

Gene expression profiles were assessed with a single-channel Agilent Tobacco Gene Expression Microarray, consisting of 43,759 probes corresponding to 26,942 unigenes (see Materials and Methods). Probe annotation was based on the Mapman ontology available at the GoMapman website resource^4^ (Ramšak et al., 2014). Although the Mapman file at this website is the most complete publicly available repository for tobacco DNA sequences, it should be noted that 30% of the probes could not be assigned to any functional category due to unknown function or ontology. The total number of leaf-expressed genes that passed the background correction and filtering processes (see Materials and Methods) was similar for the two genotypes (WT and *pfld*4-2) and represented about 72% of the total microarray unigenes.

### The Presence of Fld in Chloroplasts Affected Gene Expression Patterns in Tobacco Leaves

We first analyzed changes in transcript levels caused by Fld expression under mock conditions to identify pathways that might respond to plastid-dependent redox-based regulation. A total of 516 genes were found to be up-regulated by at least twofold in *pfld*4-2 leaves relative to WT siblings, 91 of which increased fourfold or more (**Figure 1A** and **Supplementary Table S2**). Similarly, the levels of 595 transcripts in Fld-expressing leaves declined to 50% or less of those measured in the wild type, 37 of which were equal or below 25% (**Figure 1A** and **Supplementary Table S2**).

Functional enrichment analysis of these DE transcripts (see Materials and Methods) revealed that among the 516 genes differentially induced in *pfld*4-2 plants there was a striking over-representation of transcripts related to protein degradation via the proteasome (**Figure 1B** and **Supplementary Table S2**). Out of 98 leaf-expressed genes associated with this system, 48 were induced by Fld expression (**Supplementary Table S2**). Their identity and possible contributions to PTI/ETI will be described in detail when reporting cluster analysis (see below). Other induced genes enriched in this group encoded proteins associated to complex I of the mitochondrial electron transport chain (corresponding to five isoforms of the mitochondrial protein prohibitin), and seven different glutathione *S*-transferases (**Figure 1B** and **Supplementary Table S2**).

Transcripts down-regulated by Fld included several genes responsive to ethylene, among them one isoform of 1-aminocyclopropane-1-carboxylate synthase (ACS), two isoforms of 1-aminocyclopropane-1-carboxylate oxidase (ACO) and 11 transcription factors of the *apetala2*/ethylene responsive (ER) family (**Supplementary Table S2**). Various genes associated with sugar and nutrient signaling were also repressed by Fld, including two genes coding for putative scopoletin glucosyltransferases, three genes coding for members of the photoassimilate-responsive protein (PAR) family, and four EXORDIUM-like transcripts (**Figure 1B** and **Supplementary Table S2**). Noteworthy, Fld-dependent down-regulation affected several genes related to biotic stress, including nine PR proteins (a thionin, a thaumatin and several chitinases and PR-1 proteins), and 13 transcription factors of the WRKY family (Ogata et al., 2012, 2013; Adachi et al., 2015).

In conclusion, Fld presence led to altered expression of about 1,100 genes in mock-inoculated tobacco plants grown under greenhouse conditions. Since the microarray analysis detected ∼18,800 transcripts in leaf tissue, the subset of leaf-expressed genes affected by Fld via alterations in chloroplast redox status and/or ROS levels was 5.8%, which represents a significant fraction of the tobacco genome.

### Inoculation of Tobacco Leaves with *Xcv* Led to Widespread Transcriptional Reprogramming in Both WT and Fld-Expressing Plants

Changes in gene expression caused by *Xcv* infiltration were determined at 19 hpi in WT and *pfld*4-2 plants, and transcripts displaying twofold or higher differences in tissues inoculated with the microorganism (compared to mock samples) were regarded as DE. The overlap of DE transcripts between treatments and plant genotypes is summarized in **Figure 2** as Venn diagrams. Analysis of the overall results indicated that two thirds of DE genes were shared by the two genotypes, whereas the remaining one third were DE in WT or *pfld*4-2 leaves (**Figure 2**). Of the 698 genes exclusively induced in WT plants by the pathogen, 27% (187 genes) were already up-regulated by Fld expression under mock conditions. Likewise, of the 600 genes exclusively repressed in WT plants by the pathogen, 17% (102 genes) were already down-regulated by Fld under mock conditions. We introduce herein the term “Fld priming” for those *Xcv*-dependent responses that were already triggered by Fld in the absence of the microorganism, and presumably help the plant to better cope with the pathogen challenge.

**FIGURE 3:**
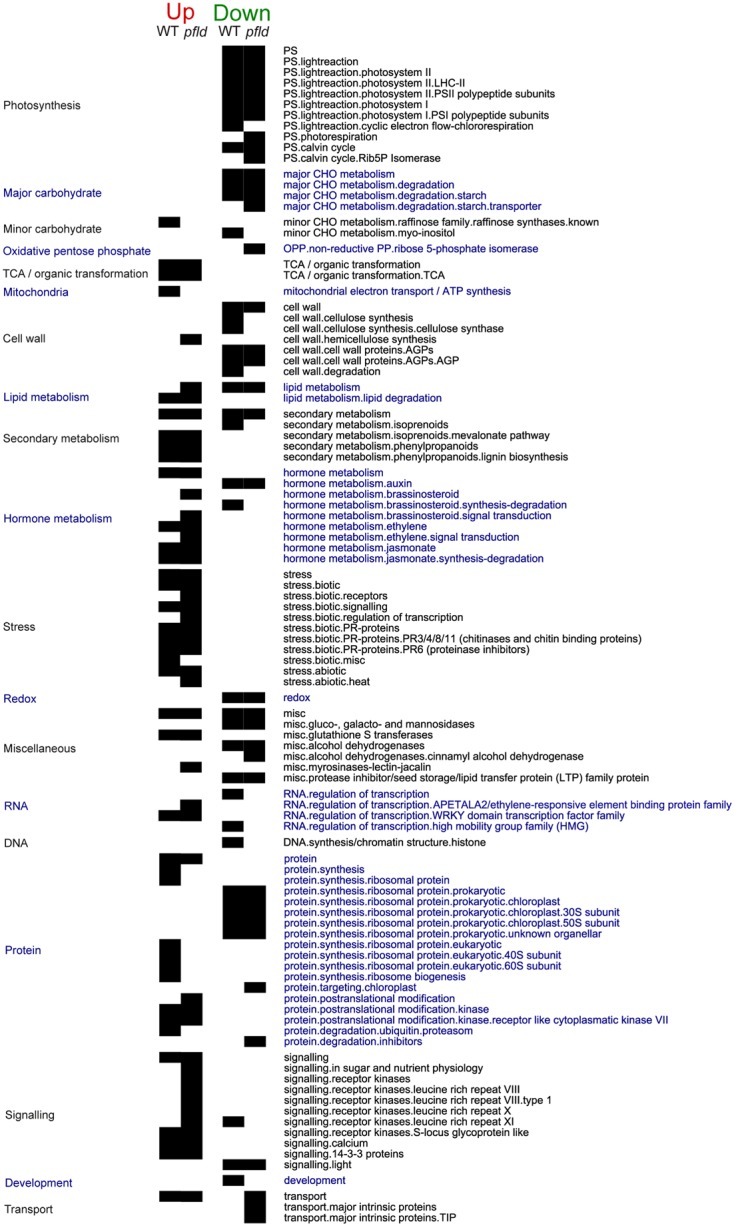
Analysis of over-represented DE genes in Mapman functional pathways during the response of *pfld*4-2 and WT genotypes to *Xcv* infiltration. The analysis was carried out separately for induced (FC > 2 and FDR < 0.05) and repressed (FC < 0.5 and FDR < 0.05) genes (Fisher’s exact test with Bonferroni correction with FDR < 0.05).

Functional enrichment analysis using Mapman identified pathways that significantly changed in response to *Xcv* inoculation in the two genotypes, including down-regulation of genes involved in photosynthesis and starch metabolism, and induction of components of the respiratory chain, the tricarboxylic acid (TCA) cycle and JA metabolism (**Figure 3**, see also cluster analysis in the following sections).

A group of DE genes were enriched in only one genotype, either WT or *pfld*4-2. Among them, 73 transcripts associated with biotic stress pathways were exclusively up-regulated by *Xcv* in *pfld*4-2 plants (**Figure 3**, see also cluster analysis in the next section). They included 12 genes whose products are involved in ethylene metabolism and regulation, six of them transcriptional regulators of the *apetala2*/ER family. It is worth noting that many of these DE genes were repressed by Fld in the absence of the microorganism, as reported before (**Figure 1B** and **Supplementary Table S2**).

*Xanthomonas campestris* pv. *vesicatoria* inoculation affected expression of genes associated with cell wall metabolism, but the effects were different between genotypes (**Figure 3**). Infection of WT plants resulted in lower levels of transcripts encoding cellulose synthase, whereas infiltration of *pfld*4-2 leaves led to increased expression of genes related to hemicellulose synthesis. The results suggest that the presence of Fld would favor hardening of the cell wall in response to the microorganism (**Figure 3**).

Other differences in the behavior of WT and *pfld*4-2 plants exposed to *Xcv* include the induction of proteasome-associated genes in WT plants but not in the transgenic line (**Figure 3**). These are genes that were constitutively activated by Fld in the absence of *Xcv* inoculation (**Figure 1B** and **Supplementary Table S2**). Indeed, proteasome-related genes represent the most remarkable example of plant priming by Fld, as defined before.

*Xanthomonas campestris* pv. *vesicatoria* challenge caused induction of 157 genes involved in biotic stress responses in both genotypes, but the transcriptional profiles exhibited significant differences between WT and *pfld*4-2 plants (**Supplementary Table S3**). PR proteins (especially those belonging to the PR-1, 2, 3, 4, 6, 8, 11, and 12 classes) and components of the biotic response signaling machinery (including homologs of the calcium-binding protein CML19, jasmonate receptor JAZ protein TIFY 10, the defense activator EDS1, U-box containing proteins, G-protein-coupled receptors of the MLO family, the SA glucosuyltransferase SGT1, and the bHLH transcription factor AIG1) were enriched in both genotypes among *Xcv*-induced genes. In contrast, transcripts encoding receptors (such as many leucine-rich repeat receptor kinases) and transcriptional regulators involved in such responses (including members of the previously mentioned ERF and WRKY families) were only enriched in the transgenic line (**Figure 3** and **Supplementary Table S3**).

### Cluster Analysis

Cluster analysis allows the identification of groups of DE genes that display similar expression patterns in response to treatment and/or genotype, suggesting that they might share common regulatory pathways. Leaf-expressed genes were grouped into 30 distinct clusters of widely different size. By far, the most densely populated cluster, containing 13,313 members and representing 71% of all leaf-expressed genes, corresponded to those transcripts which were neither affected by genotype nor treatment. The remaining genes were DE in response to the presence of Fld and/or *Xcv* and were included in the other 29 clusters. We focused on 14 clusters considered the most relevant because they were either highly populated or enriched in DE genes related to stress responses. Clusters displaying contrasting behaviors (e.g., induction vs. repression) were compared *vis-à-vis*. The complete distribution of DE genes in these 14 clusters can be found in **Supplementary Tables S4**–**7**, and the responses of DE genes included in the remaining 15 clusters (15 to 29) are described in Supplementary Figure S1.

### Regulation of Most *Xcv*-Responsive Genes Was not Affected by Fld Expression

**FIGURE 4:**
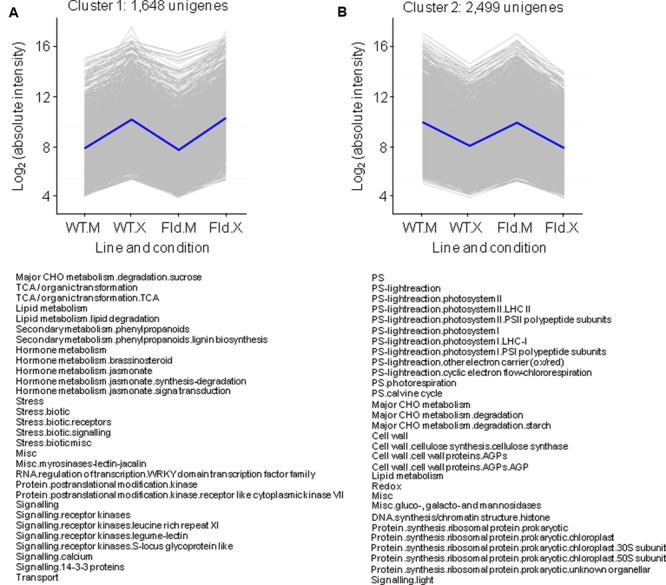
Clusters formed by *Xcv*-induced **(A)** or *Xcv*-repressed **(B)** genes independently of genotype. Each gray line of the charts corresponds to a particular gene, and the dark line represents the average behavior of all the genes contained in each cluster. Labelings in the abscissa correspond to the WT line infiltrated with mock solution (WT.M) and *Xcv* (WT.X), and the *pfld*4-2 line infiltrated with mock (Fld.M) and *Xcv* (Fld.X); those in the ordinates correspond to the absolute intensity values in log_2_ scale. For each pairwise comparison between lines or treatment, genes were defined as induced when FC > 2 and FDR < 0.05, and repressed when FC < 0.5 and FDR < 0.05. The total number of genes in each cluster is indicated above the corresponding panel, and the list of pathways with over-represented DE genes is shown below (analyzed using Fisher’s exact test with Bonferroni correction with FDR < 0.05). The list of unigenes belonging to these clusters and their corresponding descriptions, pathway assignments and fold-change values in the multiple comparison tests are described in **Supplementary Table S4**.

Clusters comprising genes that exhibited the same response to *Xcv* inoculation irrespective of the genotype accounted for ∼75% of total DE genes, with 1,648 and 2,499 transcripts that increased or decreased twofold or more relative to mock-treated samples (clusters 1 and 2, respectively, **Figures 4A,B** and **Supplementary Table S4**). Cluster 1 was enriched in genes whose products participate in the TCA cycle, in biotic stress responses and in JA synthesis, whereas cluster 2 includes genes associated with photosynthesis, starch metabolism and chloroplast protein synthesis (**Figure 4**). *Xcv*-dependent repression affected genes involved in virtually all aspects of photosynthesis, including photosystems I and II of the PETC, components of the Calvin cycle and photorespiration (**Figure 4B**).

In a context of photosynthetic decline, energy supply during pathogen infection can be compensated by increasing carbohydrate degradation and respiratory metabolism (Essmann et al., 2008). Indeed, *Xcv* inoculation had a profound effect on leaf carbohydrate metabolism, summarized as Mapman representations in Supplementary Figures S2, S3. We have previously shown that cell wall invertases (CWI), which cleave sucrose into fructose and glucose, were induced by *Xcv* in both genotypes at the transcript and activity levels (Zurbriggen et al., 2009). Induction was confirmed in this study, as three genes coding for CWI (Nta.3537, Nta.3772, and Nta.3817) could be grouped in cluster 1 (**Figure 4A** and **Supplementary Table S4**). In fact, 29 genes associated with the sucrose degradation pathway (which includes invertases) were enriched in cluster 1 (**Figure 4A** and Supplementary Figure S2 and **Table4**). They included two sucrose synthases (catalyzing sucrose hydrolysis), fructokinase 7 and α–1,3/1,6 mannosyltransferase (**Supplementary Table S4**). Conversely, genes involved in sucrose synthesis, such as fructose-1,6-bisphosphatase and three sucrose-phosphate synthase isoforms were repressed by *Xcv* inoculation and grouped in cluster 2 (**Supplementary Table S4**).

Sucrose hydrolysis is expected to provide substrates for the cytosolic branch of glycolysis, and 10 genes encoding components of this route were induced by *Xcv* in both WT and Fld-expressing plants: three isoforms of phosphoglycerate kinase, two of phosphofructokinase, two of enolase and one each of glyceraldehyde 3-phosphate dehydrogenase, aldolase and phosphoglycerate mutase (**Supplementary Table S4**). Six additional genes associated with glycolysis were induced by Fld in the absence of treatment and in some cases still further after *Xcv* inoculation, including two more pyruvate kinase isoforms, enolase, aldolase and two pyruvate carboxykinases (**Supplementary Table S2**).

Glycolysis, in turn, supplies acetyl-CoA to the TCA cycle, also enriched in cluster 1 (**Figure 4A** and Supplementary Figure S3). Genes coding for most of the enzymes of TCA cycle are part of this cluster, including a citrate synthase, a regulatory subunit of the isocitrate deshydrogenase, four components of the 2-oxoglutarate dehydrogenase complex, a succinyl-CoA ligase, a succinate dehydrogenase subunit and a fumarase (**Supplementary Table S4**).

While many genes included in the functional category Mitochondrial Electron Transport/ATP synthesis were up-regulated by Fld expression and/or *Xcv* infiltration (**Supplementary Table S4**), most of them, including prohibitin isoforms and alternative oxidoreductases, do not function in the central pathway of respiratory electron transfer, except for three subunits of the cytochrome *b*-*c1* complex and one of the cytochrome *c* oxidase, which were induced by the microorganism in WT plants but not in the transgenic line (**Supplementary Table S4**). In the case of the ATP synthesis machinery, a gene coding for the 𝜀 subunit was part of cluster 1 (**Supplementary Table S4**), while expression of the other subunits was not modified.

Cluster 1 was also enriched in genes related to hormone function (**Figure 4A** and **Supplementary Table S4**). SA, JA, and ethylene are integral components of signal transduction networks involved in activation of plant innate immunity. We have previously reported that *Xcv* inoculation led to similar increases of JA levels in *pfld*4-2 and WT plants (Zurbriggen et al., 2009). In good agreement with those observations, genes related to JA metabolism were over-represented in cluster 1 (**Figure 4A**), including DE genes involved in both JA synthesis (coding for several lipoxygenases, oxophytodienoate reductases, a phospholipase A1, a divinyl ether synthase, and an allene oxide synthase), as well as different types of JA receptor JAZ proteins (TIFY10) (**Supplementary Table S4**). Similar results had been obtained by the functional enrichment analysis of *Xcv* responses (**Figure 3**).

Other hormones have also been implied in plant biotic interactions, in some cases through modulation of host defense responses initiated by the SA–JA–ethylene systems. For instance, pathogen infection has been reported to induce brassinosteroid synthesis and signaling pathways (Nakashita et al., 2003). Cluster analysis showed that several genes associated with brassinosteroid metabolism were strongly induced by *Xcv* infiltration in plants of both genotypes (cluster 1 in **Figure 4A** and **Supplementary Table S4**). They included a BEH4-like transcription factor (Nta.10658) and a BAK1-like kinase (Nta.11817). However, genes involved in brassinosteroid signaling were only over-represented among those induced in *pfld*4-2 leaves (**Figure 3** and **Supplementary Table S2**).

Most inducible defense proteins have been classified as PR proteins, divided into 17 classes (PR-1 to PR-17) based on their biological activities, physicochemical properties and sequence similarities (van Loon et al., 2006). These proteins do not constitute a superfamily but rather a collection of structurally and functionally unrelated proteins commonly involved in defense, including chitinases and glucanases, proteinase inhibitors and antimicrobial peptides such as thionins and defensins (van Loon et al., 2006). We have previously reported that WT and *pfld*4-2 plants exhibited a strong induction of the PR genes coding for PR-Q, SAR8.2 and PR-1b upon *Xcv* infection (Zurbriggen et al., 2009). Data obtained by microarray analysis confirmed those observations, as the three groups of genes showed strong induction by *Xcv*. All genes coding for SAR8.2 isoforms (Nta.7747, Nta.9455, Nta.5457, Nta.319, and Nta.2644) were found in cluster 1 (**Figure 4A**). This cluster harbored a total of 41 PR genes and was the most densely populated by PR proteins, including chitinases of the PR-3 and PR-4 classes and proteinase inhibitors of the PR-6 class (**Supplementary Table S4**).

With respect to *Xcv*-repressed genes, functional analysis revealed an over-representation of genes associated with redox metabolism in both genotypes (**Figure 3**), and this pattern was reflected by enrichment of DE genes related to these pathways in cluster 2 (**Figure 4B**). They encoded peroxiredoxins, glutaredoxins, thioredoxins and enzymes of glutathione and ascorbate metabolism. Mostly plastidic isoforms were affected (**Supplementary Table S4**). The results suggest that there was an overall down-regulation of scavenging systems upon *Xcv* infection, as already reported for other plant-pathogen interactions (Torres et al., 2006). This decline was not prevented by Fld, a chloroplast antioxidant *per se* (Zurbriggen et al., 2009). On the other hand, there were six different glutathione *S*-transferases among Fld-induced genes under control conditions (**Figure 1B**), which were further induced by *Xcv* inoculation (**Figure 3**). These enzymes catalyze the conjugation of glutathione to a variety of hydrophobic substrates, such as toxins and radical-chain reaction byproducts, rendering them less reactive and more water-soluble, and thus more tractable for vacuolar sequestration (Dixon and Edwards, 2010). Induction of conjugative systems via glutathione *S*-transferases in *pfld*4-2 plants could provide an alternative mechanism of ROS detoxification.

Biphasic ROS production in the apoplast, mediated by plasma membrane NADPH oxidase activities encoded by the *Rboh* gene family, has been long considered as a central feature of successful pathogen recognition (Morales et al., 2016). *RbohD* (unigene Nta.3862) showed a strong induction by *Xcv* in both genotypes and thus classified in cluster 1 (**Supplementary Table S4**), while expression of other genes of the *Rboh* family (Nta.3815, Nta.4371, Nta.2526) were not affected by either pathogen or Fld. Microscopic analysis of *Xcv*-inoculated leaves treated with a ROS-dependent fluorescent probe revealed that ROS build-up was largely abolished in chloroplasts of *pfld*4-2 tissues, whereas apoplastic ROS accumulation was unaffected (Zurbriggen et al., 2009).

### Genes Coding for the Two Photosynthetic Fd Isoforms were Down-Regulated under *Xcv* Infection

Among the genes repressed by *Xcv* in both WT and *pfld*4-2 plants were those encoding the tobacco photosynthetic Fds (unigenes Nta.2519 and Nta.9424, **Supplementary Table S4**). They are particularly interesting because Fd is the universal electron acceptor of the PETC and the isofunctional counterpart of Fld. We have previously reported that total Fd protein levels were down-regulated in WT plants infected with *Xcv* compared to mock-infiltrated siblings (Zurbriggen et al., 2009).

Fd expression is repressed under most conditions of abiotic stress (Hruz et al., 2008), and the stress protection conferred by Fld can be explained, at least in part, by functional complementation of stress-labile Fd in both photosynthetic microorganisms (Chappell and Webb, 2010; Thompson et al., 2011) and transgenic plants (Tognetti et al., 2006; Blanco et al., 2011). The fate of Fd during episodes of biotic stress is instead less clear. Of 30 biotic interactions reported for Arabidopsis leaves in Genevestigator (Hruz et al., 2008), nine different microorganisms and strains caused down-regulation of at least one of the two photosynthetic isoforms of Fd (AtFd1 or AtFd2), only one interaction showed induction of AtFd1, and the rest displayed no significant changes (**Table 1**). Photosynthetic Fds were also repressed when infiltrated with the PAMP flg22 (**Table 1**), a peptide derived from the bacterial flagellin N-terminus, while remaining unaffected in other four PAMP leaf treatments (data not shown). At the protein level, leaf Fd contents were shown to decline in tobacco after inoculation with virulent *Pectobacterium carotovorum* subsp. *carotovorum* (Huang et al., 2007) and tobacco mosaic virus (Ma et al., 2008). Besides photosynthetic Fds, plants contain additional Fd isoforms involved in nitrite reduction and other assimilatory pathways (e.g., AtFd3 of Arabidopsis). These isoforms are preferentially expressed in non-photosynthetic tissues such as roots, but they are also present at lower levels in leaves (Hanke et al., 2004), and one unigene (Nta.7381) with high sequence identity with AtFd3 could be retrieved in our leaf microarray assay. Interestingly, this Fd isoform was also regulated by *Xcv* inoculation, but in the opposite direction to that exhibited by its photosynthetic counterparts: it was induced ∼5fold upon *Xcv* infiltration in both WT and *pfld*4-2 plants (cluster 1 in **Figure 4A** and **Supplementary Table S4**). Analysis of the expression of heterotrophic Fds in biotic stress situations indicated that they were induced in 12 different interactions (**Table 1**), while no changes could be detected in other 18 (data not shown). Our results then indicate that repression of photosynthetic Fds and induction of the heterotrophic isoforms are commonplace during plant biotic interactions.

**Table 1:** Analysis of Fd expression in Arabidopsis leaves during different biotic interactions.

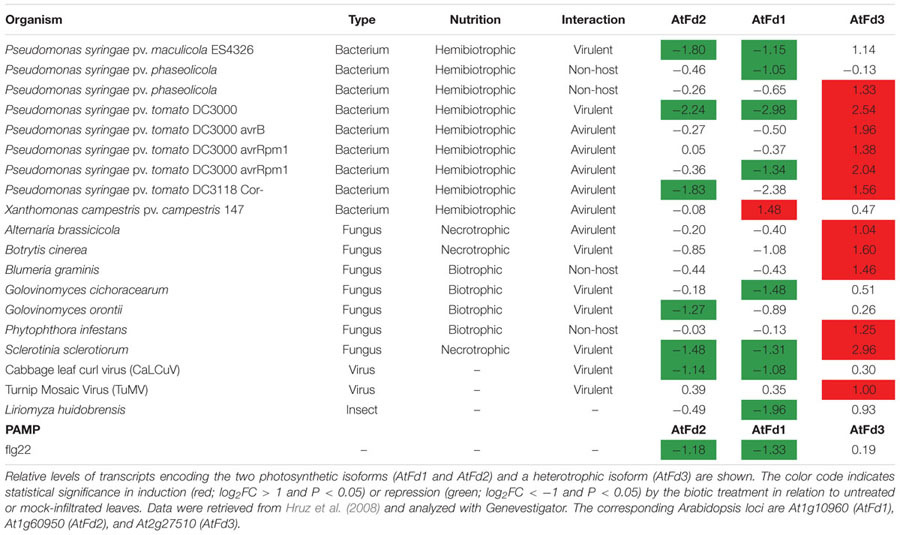

### Genes Whose *Xcv* Responses were Modulated by Fld Expression

**FIGURE 5:**
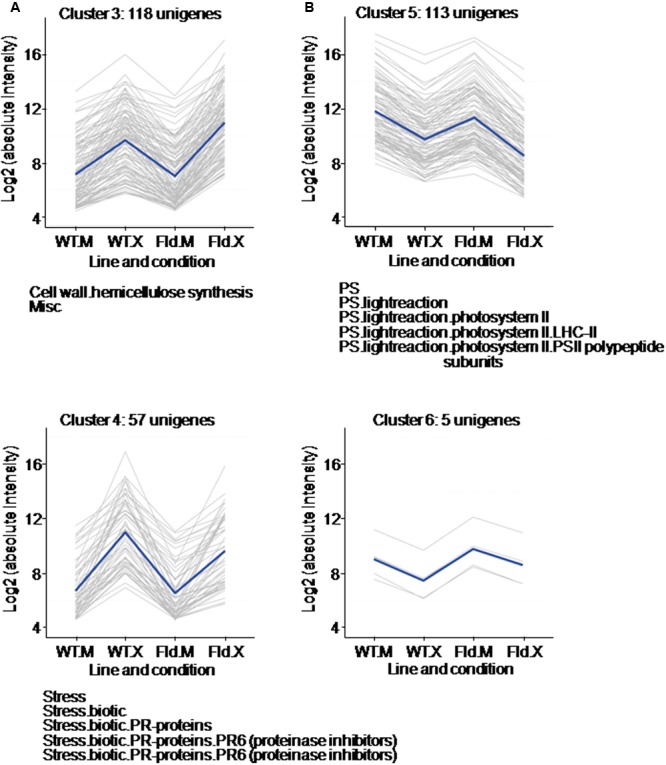
Clusters formed by *Xcv*-induced **(A)** or *Xcv*-repressed **(B)** genes, in which the responses to the microorganism were exacerbated or ameliorated by Fld expression. Each gray line of the charts corresponds to a particular gene, and the dark line represents the average behavior of all genes of the cluster. Labelings of the abscissa and ordinates, total number of genes in each cluster and list of pathways with over-represented DE genes are indicated as in **Figure 4**. Further details are given in **Supplementary Table S5**.

Among the DE genes whose *Xcv* responses were affected by the presence of Fld, a first group of two clusters (3 and 4) was made up of genes that were up-regulated after *Xcv* inoculation and further affected by Fld (**Figure 5A** and **Supplementary Table S5**). Cluster 3 had 118 genes that were induced by the pathogen in both WT and *pfld4*-2 plants, but significantly more in the transgenic line (**Figure 5A**). Over-represented in this cluster were DE genes associated with hemicellulose synthesis. *Xcv* inoculation resulted in up-regulation of 17 genes involved in this pathway in WT plants, and four additional genes in the transgenic line (**Supplementary Table S4**). The results suggest that hardening of the cell wall is favored by Fld expression during this biotic interaction.

Cluster 4, in turn, contained 57 genes whose *Xcv*-dependent induction was decreased by Fld (**Figure 5A** and **Supplementary Table S5**). This cluster was enriched in transcripts associated with biotic stress responses, especially PR-6 proteins with proteinase inhibitor activities (**Supplementary Table S5**). While expression of numerous PR proteins was induced by *Xcv* in WT and *pfld*4-2 plants, therefore classifying in cluster 1 (**Figure 4A**), cluster 4 was the one with the highest proportion of PR genes (10 out of 57), and most of them were members of the PR-6 subclass (**Supplementary Table S5**).

A second group of two clusters (5 and 6) contained DE genes down-regulated by *Xcv*. Cluster 5 included 113 genes that were repressed by *Xcv* in both genotypes, but significantly more in *pfld*4-2 plants (**Figure 5B**). A large fraction of these DE genes encodes components of the photosynthetic machinery, including six different chlorophyll *a*/*b* binding proteins, three subunits of PSI, one subunit of PSII, one isoform of the Rubisco small subunit, a photosynthetic NDH subunit of subcomplex B5, and a Rubisco large subunit-binding protein (**Supplementary Table S5**). The cluster displaying the opposite pattern of expression, whose *Xcv*-dependent repression was ameliorated by Fld, was sparsely populated with only 5 unigenes (cluster 6 in **Figure 5B**).

In conclusion, the presence of Fld exacerbated rather than attenuated *Xcv* responses (231 genes vs. 62 genes), suggesting that the presence of the flavoprotein allowed the plant to deploy a more complete and varied response against the attacking microorganism.

### *Xcv-*Responsive Genes Primed by Fld Expression in the Absence of Infection

**FIGURE 6:**
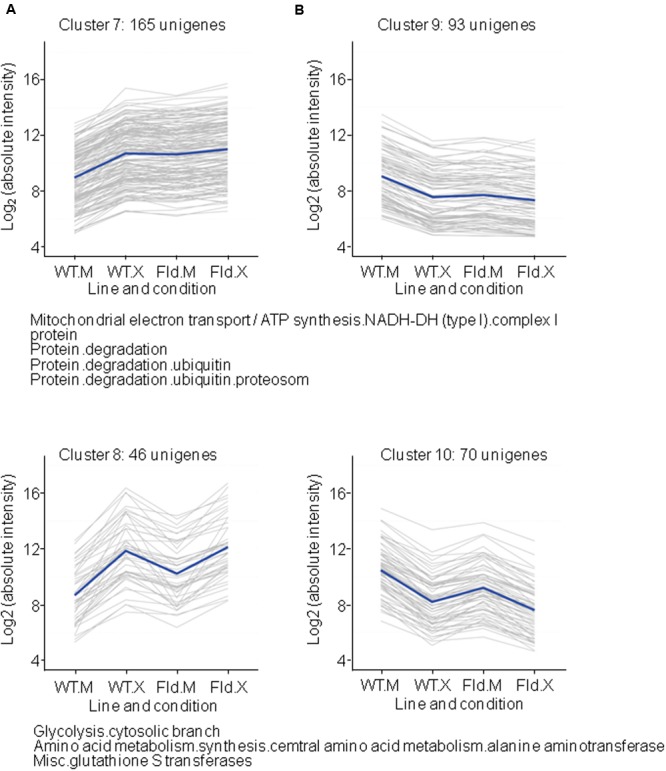
Clusters formed by *Xcv*-induced **(A)** or *Xcv*-repressed **(B)** genes, which were already primed by Fld under control conditions. Each gray line of the charts corresponds to a particular gene, and the dark line represents the average behavior of all genes of the cluster. Labelings of the abscissa and ordinates, total number of genes in each cluster and list of pathways with over-represented DE genes are indicated as in **Figure 4**. Further details are provided in **Supplementary Table S6**.

Another group of clusters is represented by those *Xcv*-responsive genes whose expression was fully or partially modulated by Fld in the absence of infection and in the same direction as pathogen exposure (**Figure 6** and **Supplementary Table S6**). Up-regulated genes were divided into two clusters depending on the degree of induction by Fld and *Xcv*. Cluster 7 included those genes which were induced by Fld to the same levels that they reached in *Xcv*-inoculated WT plants, and were not further affected by exposure to the microorganism (**Figure 6A**). Genes belonging to this cluster are largely associated with protein degradation via the proteasome, and with different prohibitin isoforms linked to complex I of the mitochondrial electron transport chain (**Supplementary Table S6**). Cluster 8 included DE genes that were partially activated by the flavoprotein in the absence of the microorganism, but only attained maximal expression levels upon *Xcv* inoculation, without major differences between genotypes. Genes coding for additional isoforms of glycolytic enzymes and glutathione *S*-transferases were over-represented in this cluster (**Figure 6A** and **Supplementary Table S6**). Finally, DE genes belonging to clusters 9 and 10 were repressed by Fld, this trend being maintained or even exacerbated by *Xcv* challenge (**Figure 6B** and **Supplementary Table S6**).

A total of 221 DE genes could be grouped in clusters 7 and 8. The most remarkable observation was the generalized induction of components of the 26S proteasome (26SP) by Fld, even in the absence of infection (**Figures 1B, 6A** and Supplementary Figure S4). The 26SP complex is a multi-subunit, multi-catalytic protease responsible for most cytosolic and nuclear protein turnover, and composed of two sub-particles, the 19S regulatory particle (19SRP) that binds and unfolds protein targets, and the 20S core particle (20SCP) that degrades proteins into small peptides (Geng et al., 2012). Most 26SP substrates are conjugated to a poly-ubiquitin (Ub) chain that serves as a degradation signal. However, some targets, such as oxidized proteins, do not require a poly-Ub tag for proteasomal degradation, and recent studies have shown that the main protease in this Ub-independent pathway is free 20SCP (Kurepa and Smalle, 2008). Virtually all genes encoding proteasomal subunits were induced by Fld following the same pattern, corresponding to cluster 7 in **Figure 6A**. They included components of the 20SCP such as subunits α1, α3 to α7, and β1 to β7, as well as components of the 19SRP such as RPN9, RPN1 and the chaperone UMP1 required for correct maturation of the 20SCP (Dudler, 2013, see **Supplementary Table S6**).

Expression of proteasome components was also enhanced by *Xcv* in the wild type (**Figure 6A** and Supplementary Figure S4), in line with a role of this proteolytic complex in various defense responses including oxidative stress protection and regulation of LCD networks. A recent report describing genome-wide responses of tobacco plants to different microorganisms also observed generalized induction of proteasome subunits upon leaf infiltration with two non-host *Pseudomonas syringae* pathovars preferentially eliciting PTI or ETI responses (Bozsó et al., 2016).

The role of the proteasome in biotic stress responses was also illustrated by the observation that some key plant defense signaling components, such as those involving JA, abscisic acid (ABA) and auxin signaling, showed 26SP-dependent degradation (Santner and Estelle, 2010; Lu et al., 2011). In addition, the proteasome is a direct target of bacterial effectors (Groll et al., 2008; Üstün et al., 2013), which highlights its importance in plant–microbe interactions.

Different parts of ETI- or PTI-associated signaling networks can be influenced by the proteasome. Furlan et al. (2012) proposed that this proteolytic system could regulate PTI by controlling the amounts of PAMP receptors. Indeed, the FLS2 flagellin receptor is internalized by endocytosis and degraded by the proteasome, as part of a feedback regulation of PTI that modulates the intensity and duration of resistance responses (Lu et al., 2011). Moreover, inhibition of the proteasomal signaling pathway using a pharmacological approach preferentially affected PTI responses in tobacco (Bozsó et al., 2016). Thus, our results and those of other groups (Furlan et al., 2012; Bozsó et al., 2016) strongly suggest that increased expression of the proteasome complex is a key component of a successful plant response to biotic stress. It is remarkable that this up-regulation could be mediated by chloroplast ROS and/or redox status.

### Genes Regulated by Fld and *Xcv* in Opposite Directions

**FIGURE 7:**
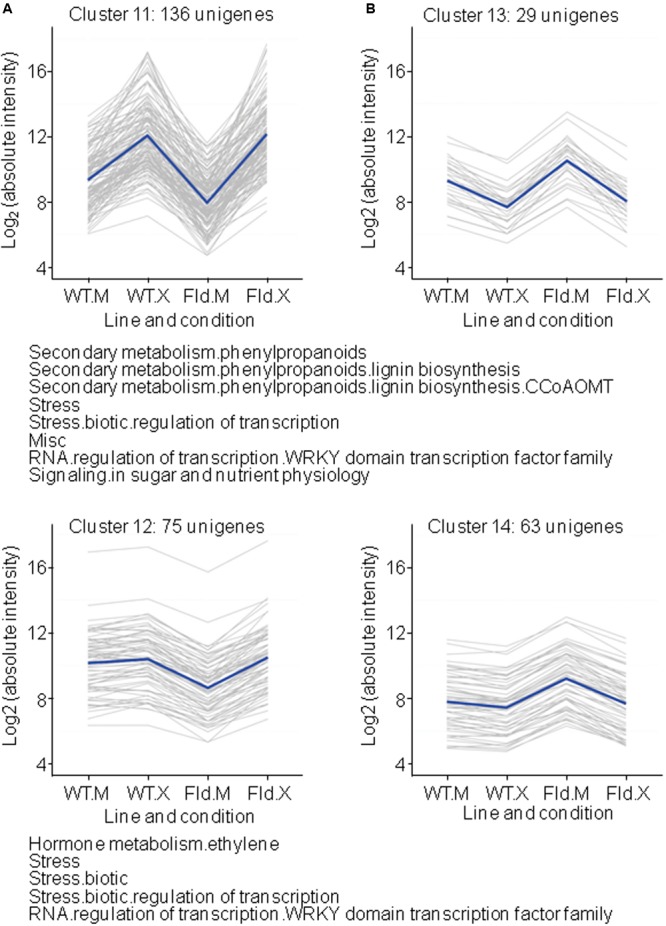
Clusters formed by Fld-repressed **(A)** or Fld-induced **(B)** genes, in which the effect of the flavoprotein was reverted by *Xcv* infection. Each gray line of the charts corresponds to a particular gene, and the dark line represents the average behavior of all genes of the cluster. Labelings of the abscissa and ordinates, total number of genes in each cluster and list of pathways with over-represented DE genes are indicated as in **Figure 4**. Further details are given in **Supplementary Table S7**.

An interesting group of clusters was populated by DE genes up- or down-regulated by Fld under mock conditions, with this effect being reverted, to various extents, after *Xcv* infiltration (**Figure 7** and **Supplementary Table S7**). Clusters 11 and 12 included genes whose *Xcv* responses were initiated from a lower expression level in the transgenic plants (**Figure 7A**). Among them, 136 genes were induced in both genotypes to similar final levels after *Xcv* infiltration (cluster 11), whereas 75 were induced only in *pfld*4-2 leaves to return to WT expression levels (cluster 12). Genes involved in lignin biosynthesis, ethylene metabolism and sugar and nutrient signaling, as well as biotic stress-responsive transcriptional regulators of the WRKY family were over-represented in these clusters (**Supplementary Table S7**).

As indicated before (**Figure 1B** and **Supplementary Table S2**), several genes involved in ethylene metabolism were repressed by Fld in the absence of *Xcv* (**Figure 1B**). For most of these genes, including ACS and members of the *apetala2*/ER family of transcriptional regulators, repression was reverted by *Xcv* exposure (cluster 12 in **Figure 7A**). One isoform of ACS, the enzyme catalyzing the rate-limiting step of ethylene biosynthesis (Chae and Kieber, 2005), followed this trend and classified in cluster 12 (Nta.2999), whereas two other isoforms (Nta.14789 and A_95_P034878) showed a similar pattern of *Xcv* induction in both genotypes and therefore belonged to cluster 1 (**Figure 4A**). In addition, many ethylene-responsive factors (ERF) have been implicated as LCD inducers in tobacco and other species (Ogata et al., 2012, 2013). Over-expression of the NtERF3a gene, a transcriptional repressor containing the ERF-associated amphiphilic repression (EAR) motif in the C-terminal region, resulted in HR-like cell death in tobacco (Ogata et al., 2012). This gene was induced by *Xcv* in WT and *pfld*4-2 plants, but was repressed by Fld under mock conditions (cluster 11 in **Figure 7A**). Although little is known about the involvement of ROS signaling in the regulation of ERFs, these results suggest a role of chloroplast ROS in the activation of some ERFs. Other EAR-motif-containing ERF genes with cell death-inducing ability are represented in our microarray (Ogata et al., 2013). Two of them (NtERF3a and NtERF6a) exhibited an expression pattern corresponding to cluster 11, whereas the other two (EREBP5, NtERF#111) belonged to cluster 12 (**Figure 6A**), being repressed by Fld in plants exposed to *Xcv* or the mock solution. This could explain in part the failure of Fld-expressing plants to elicit LCD upon *Xcv* infiltration (Zurbriggen et al., 2009).

As previously mentioned, various genes associated with sugar and nutrient signaling were repressed by Fld (**Figure 1B**). Some of them were induced in both genotypes to similar final levels after *Xcv* infiltration (cluster 11), including genes coding for two putative scopoletin glucosyltransferases, two members of the PAR family, a EXORDIUM-like protein and a glutamate receptor.

Other DE genes over-represented in cluster 11 are involved in lignin biosynthesis, and include genes coding for 4 caffeoyl-CoA O-methyltransferases, a cytochrome P450, an oxalate-CoA ligase and a quinone-oxidoreductase. Finally, DE genes encoding WRKY proteins were extensively represented in these clusters. The WRKY family of transcription factors is one of the largest described in plants, with over 74 members in Arabidopsis (Eulgem and Somssich, 2007). In this species, 49 out of 72 tested WRKY genes responded to bacterial infection or SA application (Dong et al., 2003). There were 102 tobacco unigenes assigned to the WRKY family in our microarray, and expression of 48 of them was affected by either Fld presence or *Xcv* treatment. Genes encoding WRKY proteins were over-represented in cluster 1 (23 unigenes), cluster 11 (nine unigenes), and cluster 12 (five unigenes). Furthermore, nine WRKY-encoding genes were part of cluster 2, one of cluster 4 and one of cluster 5. Then, the vast majority of WRKY genes (38 out of 48) were induced by *Xcv*. Sixteen of them were also modulated by Fld expression, in almost all cases counter-acting the effect of the microorganism.

Of those genes induced by Fld under mock conditions, 29 were repressed by *Xcv* inoculation in both WT and *pfld*4-2 plants to attain similar levels under stress (cluster 13 in **Figure 7B**), whereas 63 were repressed only in *pfld*4-2 leaves to recover WT expression levels (cluster 14 in **Figure 7B**). The most remarkable observation regarding these DE genes is the presence of genes coding for dicer-like (DCL) proteins in cluster 13 (**Supplementary Table S7**). DCLs are main component of the RNA silencing machine (Cao et al., 2016). The importance of RNA silencing in plant viral defense is underscored by the fact that it has elicited counter-defense measures from viruses to overcome it. Apart from viral defense, evidence accumulates for RNA silencing to play a role in plant interactions with bacterial and fungal pathogens (Cao et al., 2016).

### Comparative Expression Analysis of Selected DE Genes in WT and *pfld*4-2 Leaves

To validate the results obtained with the RNA microarray assay, and to provide further insight into the transcriptional responses elicited by WT and Fld-expressing leaves to *Xcv* inoculation, the expression of a number of DE genes was evaluated by qRT-PCR. In addition to the two genotypes (*pfld*4-2 and WT) analyzed by the microarray technique, the qRT-PCR measurements were extended to lines *pfld*5-8, *pfld*5-4 (the latter obtained by crossing *pfld*5-8 and WT plants, and therefore containing 50% Fld contents), and *cfld*1-4, which expresses high levels of Fld in the cytosol (Supplementary Figure S5). These transgenic lines were studied because phenotypic observations have shown that the effects of Fld were dose-dependent and required chloroplast location (Tognetti et al., 2006; Zurbriggen et al., 2009; Ceccoli et al., 2012).

**FIGURE 8:**
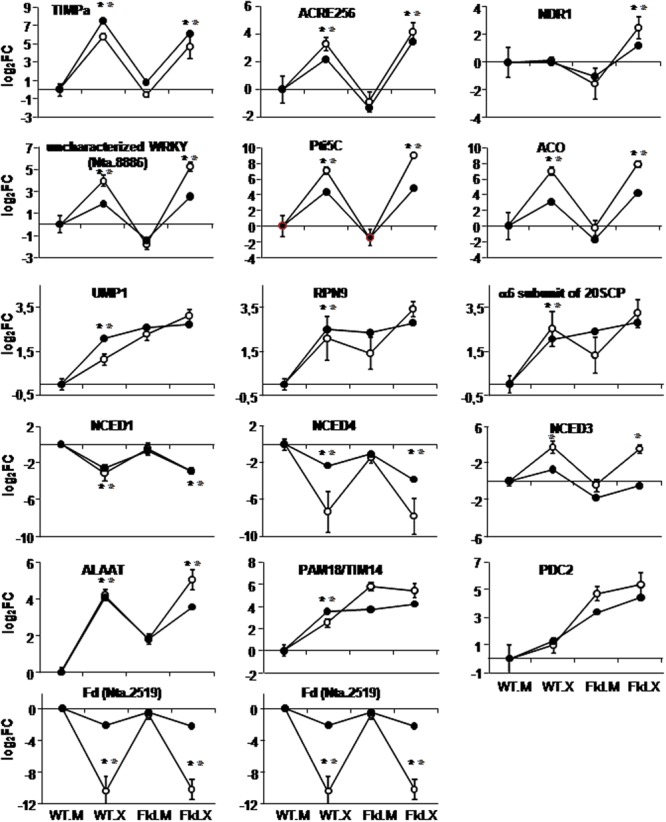
Comparison of expression patterns of selected genes as determined by microarray analysis (dark circles) and qRT-PCR (open circles). Labelings of the abscissa and ordinates are indicated as in **Figure 4**, except that fold-change values in the ordinates are represented in log_2_ scale relative to those of WT siblings infiltrated with mock solution (WT.M). Each data point of qRT-PCR determinations represents the mean and standard deviation of 5–6 biological replicates. Values corresponding to each technique are joined by lines to facilitate visual comparison with the representations of cluster analyses, without implying functional relationships. Within each line, a statistically significant effect of the infection is indicated as a gray asterisk for microarray data (FDR < 0.05 and FC > 2 or FC < 0.5) and a black asterisk for qPCR data (*p*-values < 0.05 according to ANOVA test).

The genes studied by qRT-PCR were selected on the basis of their reported association with stress responses, or of their profiles in the microarray assay. The two photosynthetic Fds, the counterparts of Fld, were also validated. Out of 17 DE genes analyzed, nine showed a strict correlation between the microarray and qRT-PCR data, including the FC (**Figure 8**). Another seven genes displayed the same expression patterns in both procedures, but quantitative differences were observed between them in *Xcv*-infected plants of the two genotypes. In all seven cases, FCs were significantly higher in qRT-PCR compared to the microarray, although these differences did not modify cluster assignment (**Figure 8** and Supplementary Figure S5). It is worth noting, within this context, that no differences between mock-infiltrated and non-infiltrated WT leaves were obtained in the qRT-PCR experiments (Supplementary Figure S5).

As previously described, DE genes associated with biotic stress responses were over-represented in various clusters. Some of them were therefore confirmed by qRT-PCR experiments. Among the *Xcv* responses that were attenuated in *pfld*4-2 plants (cluster 4 in **Figure 5A**), there was an over-representation of genes encoding PR-6 proteins such as the serine-protease inhibitor TIMPa (Linthorst et al., 1993). Results obtained by qRT-PCR showed a good correlation with the microarray analysis (**Figure 8**). A similar coincidence between the two procedures was obtained upon validation of the ACRE256 gene (**Figure 8**), predicted to encode a rapidly elicited protein kinase (Durrant et al., 2000), and of a member of the non-race-specific disease resistance 1-like gene family (NDR1-like, Varet et al., 2003), belonging to clusters 28 and 29, respectively (Supplementary Figure S1). Two transcriptional regulators related to biotic stress responses were also validated. They were part of cluster 11 (**Figure 7A**), and encode an uncharacterized WRKY transcription factor (Nta.8886) and the ERF Pti5C (Wu et al., 2015). While expression patterns obtained by microarray analysis and qRT-PCR were qualitatively similar, the latter procedure gave significantly higher FCs in the *Xcv*-inoculated tissue for both WT and *pfld*4-2 leaves (**Figure 8**).

Two DE genes associated with hormone-dependent pathways (ethylene and ABA) were studied in detail. A gene coding for an ACO isoform was repressed by Fld, whereas *Xcv* induced its expression, especially in the transgenic plants (cluster 28 in Supplementary Figure S1). The microarray results were validated by qRT-PCR, with moderately higher FCs in the latter assay (**Figure 8**).

The rate-limiting step in the biosynthesis of ABA is catalyzed by the enzyme 9-*cis*-epoxycarotenoid dioxygenase (NCED), with several isoforms encoded by a small gene family (Finkelstein, 2013). Three members were represented in the microarray, and displayed contrasting responses to challenge with the microorganism. NCED1 was repressed by *Xcv* without any significant Fld effect, therefore conforming to cluster 2 (**Figure 4B**), NCED4 was down-regulated by both Fld and *Xcv*, and NCED3 showed the opposite behavior, corresponding to clusters 22 and 25, respectively (Supplementary Figure S1). This behavior was intriguing and worth of confirmation. Expression patterns of the three genes could be validated by qRT-PCR (**Figure 8**). NCED1 exhibited a good quantitative correlation between the two procedures, whereas NCED3 and NCED4 displayed higher FCs in qRT-PCR without change in cluster assignation (**Figure 8**).

As indicated previously, there was a remarkable induction of members of the protein degradation pathway via proteasome in plants expressing Fld (**Figure 3** and Supplementary Figure S4). Three genes belonging to this route (UMP1, the RPN9 subunit of the 19SRP complex and the α6 subunit of the 20SCP complex) were induced by Fld as represented by clusters 7 and 8 (**Figure 6A**). All three were quantitatively validated by qRT-PCR (**Figure 8**). No significant differences between mock-infiltrated and non-infiltrated leaves were observed for any of the lines assayed (Supplementary Figure S6), indicating that differential expression in *pfld*4-2 and *pfld*5-8 lines was caused by the presence of Fld and not by the infiltration procedure.

Two genes encoding alanine aminotransferase (ALAAT) isoforms were also grouped in cluster 8, being induced by both *Xcv* and Fld (**Figure 6A**). One of the DE genes was validated, rendering similar transcript accumulation patterns in the microarray and the qRT-PCR experiments (**Figure 8**).

Pam18/Tim14 is a DnaJ domain-containing subunit of protein import motors localized in the inner mitochondrial membrane, with functions in immunity and redox metabolism (Chen et al., 2013). The gene encoding this protein was constitutively induced in *pfld*4-2 leaves to the same levels elicited by *Xcv* infiltration in WT plants (cluster 7 in **Figure 6A**). While the results obtained by qRT-PCR showed a fairly good correlation with the microarray data, up-regulation by Fld reached significantly higher transcript levels than *Xcv* induction in the wild type (**Figure 8**).

Some DE genes were selected on the basis of their expression patterns, even though the function(s) of the encoded products were not directly related to biotic stress, redox-based metabolism or chloroplast function. The PDC2 gene encoding pyruvate decarboxylase 2, involved in ethanol fermentation, was strongly induced by Fld and pathogen inoculation (cluster 21 in Supplementary Figure S1). Microarray and qRT-PCR determinations of PDC2 transcripts yielded a good quantitative correlation (**Figure 8**).

As described before, transcripts encoding photosynthetic Fds (Nta.2519 and Nta.9424) declined upon *Xcv* inoculation, with patterns corresponding to cluster 2 in both cases (**Figure 4B**). While microarray and qRT-PCR determinations gave similar expression patterns, quantitative differences in FC were observed in *Xcv*-treated plants for both Fd isoforms. Once again, higher values were obtained in the qRT-PCR assay (**Figure 8**).

Taken together, validation experiments were confirmatory of the microarray data. It should be noted, in this context, that the independent line *pfld*5-8, which contained Fld levels in chloroplasts similar to those of *pfld*4-2 (Tognetti et al., 2006), yielded essentially the same results in qRT-PCR experiments, whereas the cytosolic line *cfld*1-4 behaved largely as the wild type for most assayed genes (Supplementary Figure S5). The heterozygous line *pfld*5-4 displayed intermediate patterns of expression. The results confirmed that the effects of Fld were dose-dependent and required chloroplast location (Tognetti et al., 2006; Zurbriggen et al., 2009; Ceccoli et al., 2012).

## Conclusion

To our knowledge, this study represents the most comprehensive transcriptome analysis reported to date on the response of tobacco plants to a non-host pathogen, identifying genes and metabolic pathways associated with this type of plant-microorganism interaction in a model plant species whose genomic sequence has become available only recently. In addition, our research revealed the involvement of chloroplast redox status and/or ROS production in the regulation of gene expression during normal plant development as explored by analyzing Fld-expressing plants.

Transcriptomic changes observed during *Xcv* interaction affected diverse pathways that were directly or indirectly associated with pathogen resistance, such as synthesis of PR proteins, antioxidant metabolism, cell wall reinforcement, signaling and transcriptional regulation. Fld-dependent induction of genes related to hemicellulose metabolism is expected to favor stiffening of the cell wall (**Figure 5A**). Moreover, *pfld*4-2 plants displayed primed induction of protein degradation, glycolysis and respiratory metabolism (**Figure 6A** and Supplementary Figures S3, S4) at the expense of photosynthesis (**Figure 5B**), all typical landmarks of plant-microbe interactions (Rojas et al., 2014).

Cluster analysis revealed that the presence of the flavoprotein exacerbated rather than attenuated *Xcv* responses (231 genes vs. 62 genes, **Figure 5**), in addition to 374 *Xcv*-responsive genes primed by Fld prior to pathogen challenge (**Figure 6**). The relatively high number of genes with the same regulation by Fld and *Xcv* was intriguing, suggesting that the main effect (in quantitative terms) of Fld expression was to boost plant responses against *Xcv*.

While decreased chloroplast ROS production was the most obvious consequence of Fld expression in plastids (Tognetti et al., 2006; Zurbriggen et al., 2009), it should be borne in mind that on providing alternative, productive electron sinks to the excess of reducing equivalents in the PETC, Fld not only prevents oxygen reduction and ROS formation, but also alters the redox poise of the chain, keeping it more oxidized and closer to physiological conditions. The possibility cannot be ruled out that these changes in chloroplast redox status may also contribute to modulate LCD and genetic reprogramming during pathogen interactions in addition to chloroplast ROS production. Indeed, the role played by the redox poise of the PETC in retrograde signaling from the chloroplast to the nucleus has been extensively documented (see, for instance, Chan et al., 2016).

The most remarkable effect of *Xcv* was the generalized induction of proteasome subunits (**Figure 3** and Supplementary Figure S4). Our results suggest that induction of proteasomal activities is essential for a successful plant response to invading microorganisms, and that manipulation of protein turnover could be a promising target to generate plague-resistant crops. Research is currently underway to evaluate these possibilities.

In summary, results in this article provide a rich source of novel information on the extent of gene expression reprogramming undergone by plants during interactions with microorganisms, and on the role played by chloroplast redox chemistry in signaling these changes. These data constitute the foundation for further studies to disclose the basic mechanisms that shape plant defensive responses to attempted infection.

## Author Contributions

MZ, US, M-RH, and NC designed the experiments. JJPK, MZ, FS, SS, and SH performed the experiments. JJPK carried out the bioinformatics analyses. JJPK, MZ, SS, US, M-RH, and NC analyzed the data. JJPK, MZ, SS, US, M-RH, and NC wrote the manuscript.

## Conflict of Interest Statement

The authors declare that the research was conducted in the absence of any commercial or financial relationships that could be construed as a potential conflict of interest.

## Footnotes

1 www.gomapman.org

2 http://www.R-project.org

3 https://www.ncbi.nlm.nih.gov/geo/query/acc.cgi?acc=GSE92596

4 www.gomapman.org

## Supplementary Material

TABLE S1 Primer sets used for qRT-PCR determinations.

TABLE S2 List of tobacco genes with altered expression patterns in response to Fld. Unigene ID and description, mapman pathway annotation and fold-change (FC; in log_2_ scale) are shown. The color code indicates statistical significance in induction (red; log_2_FC > 1 and FDR < 0.05) or repression (green; log_2_FC < -1 and FDR < 0.05). Genes belonging to various pathways are displayed repetitively.

TABLE S3 List of tobacco genes with altered expression patterns in response to *Xcv* in *pfla*4-2 and/or WT leaves. Unigene ID and description, mapman pathway annotation and FC (in log_2_ scale) are shown. The color code indicates statistical significance in induction (red; log_2_FC > 1 and FDR < 0.05) or repression (green; log_2_FC < -1 and FDR < 0.05). Genes belonging to various pathways are displayed repetitively.

TABLE S4 List of tobacco genes belonging to clusters formed by *Xcv*-induced or *Xcv*-repressed genes independently of genotype (**Figure 4**). Unigene ID and description, mapman pathway annotation, and FC (in log_2_ scale) for each of the four line/treatment comparisons are shown. The color code indicates statistical significance in induction (red; log_2_FC > 1 and FDR < 0.05) or repression (green; log_2_FC < -1 and FDR < 0.05). Genes belonging to various pathways are displayed repetitively.

TABLE S5 List of tobacco genes belonging to clusters formed by *Xcv* responses exacerbated or ameliorated in the presence of Fld (**Figure 5**). Unigene ID and description, mapman pathway annotation, and FC (in log_2_ scale) for each of the four line/treatment comparisons are shown. The color code indicates statistical significance in induction (red; log_2_FC > 1 and FDR < 0.05) or repression (green; log_2_FC < -1 and FDR < 0.05). Genes belonging to various pathways are displayed repetitively.

TABLE S6 List of tobacco genes belonging to clusters formed by *Xcv*-responsive genes, which were already primed by Fld in the absence of *Xcv* inoculation (**Figure 6**). Unigene ID and description, mapman pathway annotation, and FC (in log_2_ scale) for each of the four line/treatment comparisons are shown. The color code indicates statistical significance in induction (red; log_2_FC > 1 and FDR < 0.05) or repression (green; log_2_FC < -1 and FDR < 0.05). Genes belonging to various pathways are indicated repetitively.

TABLE S7 List of tobacco genes belonging to clusters formed by Fld-regulated genes in which the effect of the flavoprotein was reverted by *Xcv* infection (**Figure 7**). Unigene ID and description, mapman pathway annotation, and FC (in log_2_ scale) for each of the four line/treatment comparisons are shown. The color code indicates statistical significance in induction (red; log_2_FC > 1 and FDR < 0.05) or repression (green; log_2_FC < -1 and FDR < 0.05). Genes belonging to various pathways are indicated repetitively.
