# Supplementary material for: Chloroplast Redox Status Modulates Genome-Wide Plant Responses during the Non-host Interaction of Tobacco with the Hemibiotrophic Bacterium Xanthomonas campestris pv. vesicatoria
Source: Front Plant Sci. 2017 Jul 4;8:1158. doi: 10.3389/fpls.2017.01158 (PMC5495832; doi:10.3389/fpls.2017.01158)
Supplement: Supplementary file 8 [file Image_1.PDF]

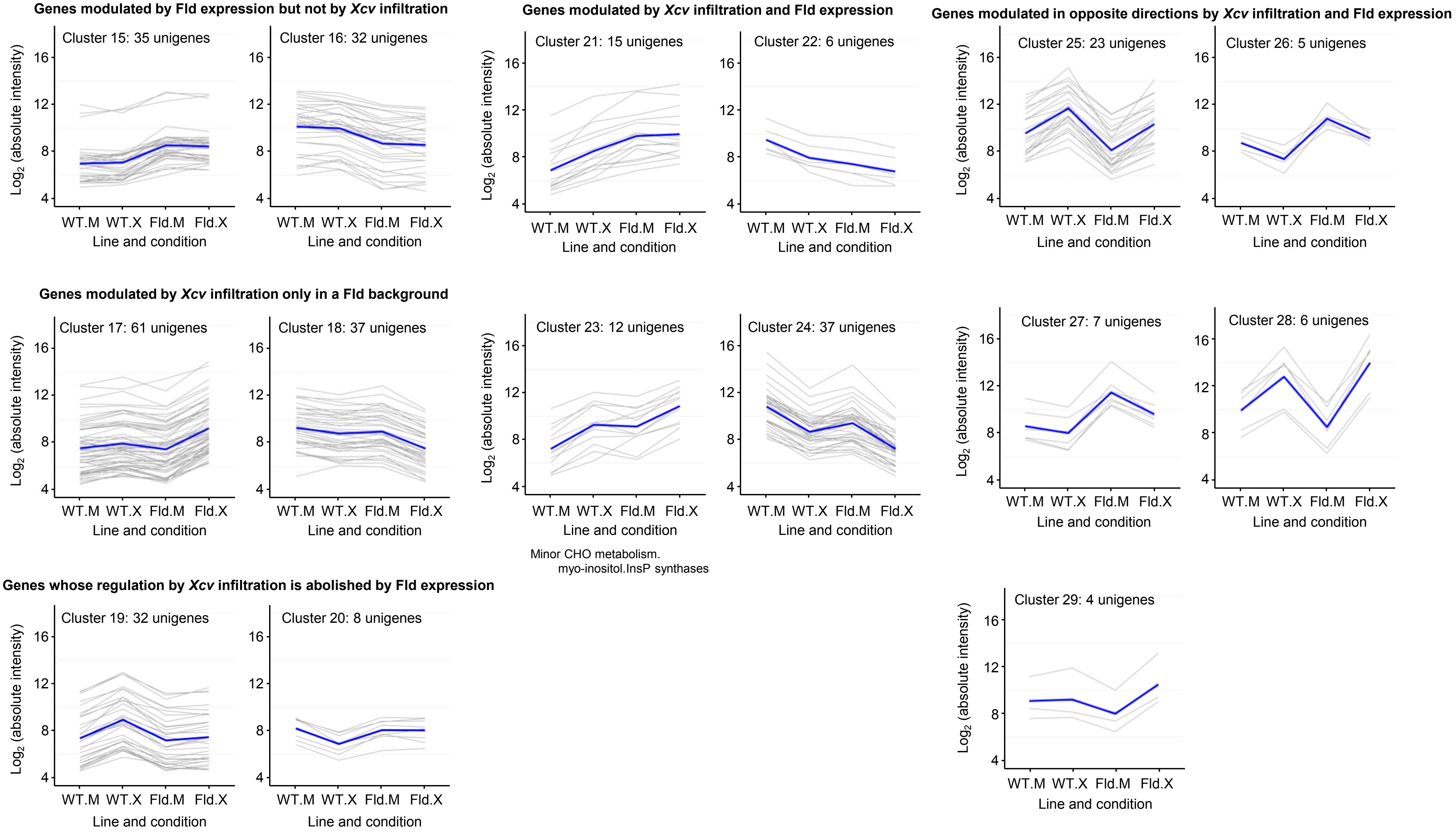

Supplementary Figure 1: Clusters not shown in the main text Figures. Each gray line of the charts corresponds to a particular gene, and the dark line represents the average behavior of the genes for the cluster. Labelings of the abscissa and ordinates and total number of genes in each cluster are indicated as in the legend to Figure 4. Only cluster 23 has a pathway with statistically over-represented DE genes.
