# Supplementary material for: Chloroplast Redox Status Modulates Genome-Wide Plant Responses during the Non-host Interaction of Tobacco with the Hemibiotrophic Bacterium Xanthomonas campestris pv. vesicatoria
Source: Front Plant Sci. 2017 Jul 4;8:1158. doi: 10.3389/fpls.2017.01158 (PMC5495832; doi:10.3389/fpls.2017.01158)
Supplement: Supplementary file 10 [file Image_3.PDF]

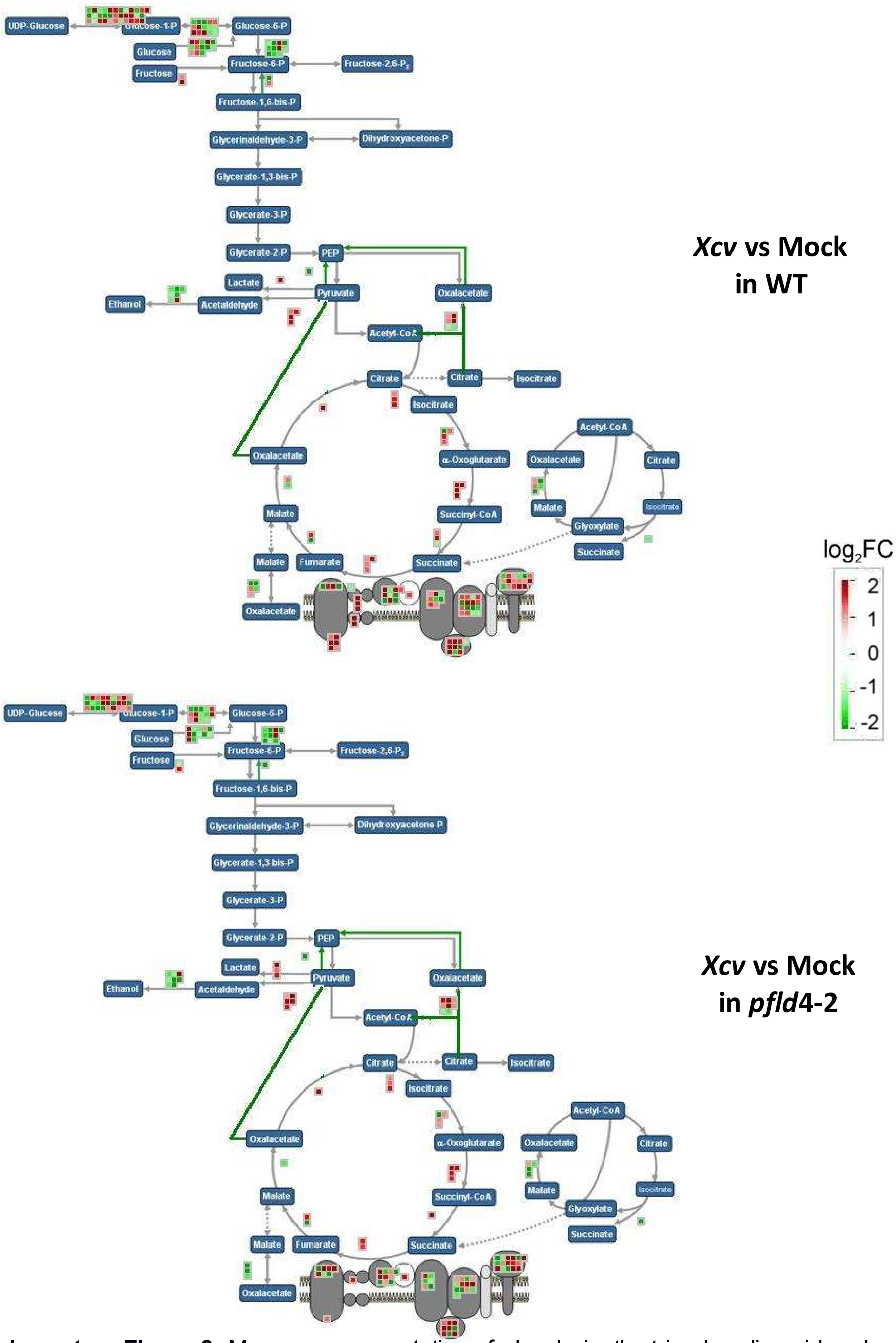

Supplementary Figure 3: Mapman representation of glycolysis, the tricarboxylic acid cycle and the mitochondrial electron transport chain showing the effect of *Xcv* infection in both genotypes. Each colored square represents a gene that is differentially induced (red; FC>2 and FDR<0.05) or repressed (green; FC<0.5 and FDR<0.05) by *Xcv* inoculation. Only DE genes are displayed. The color scheme is shown in a rectangle using a log_2 scale.
