# Supplementary material for: Chloroplast Redox Status Modulates Genome-Wide Plant Responses during the Non-host Interaction of Tobacco with the Hemibiotrophic Bacterium Xanthomonas campestris pv. vesicatoria
Source: Front Plant Sci. 2017 Jul 4;8:1158. doi: 10.3389/fpls.2017.01158 (PMC5495832; doi:10.3389/fpls.2017.01158)
Supplement: Supplementary file 12 [file Image_5.PDF]

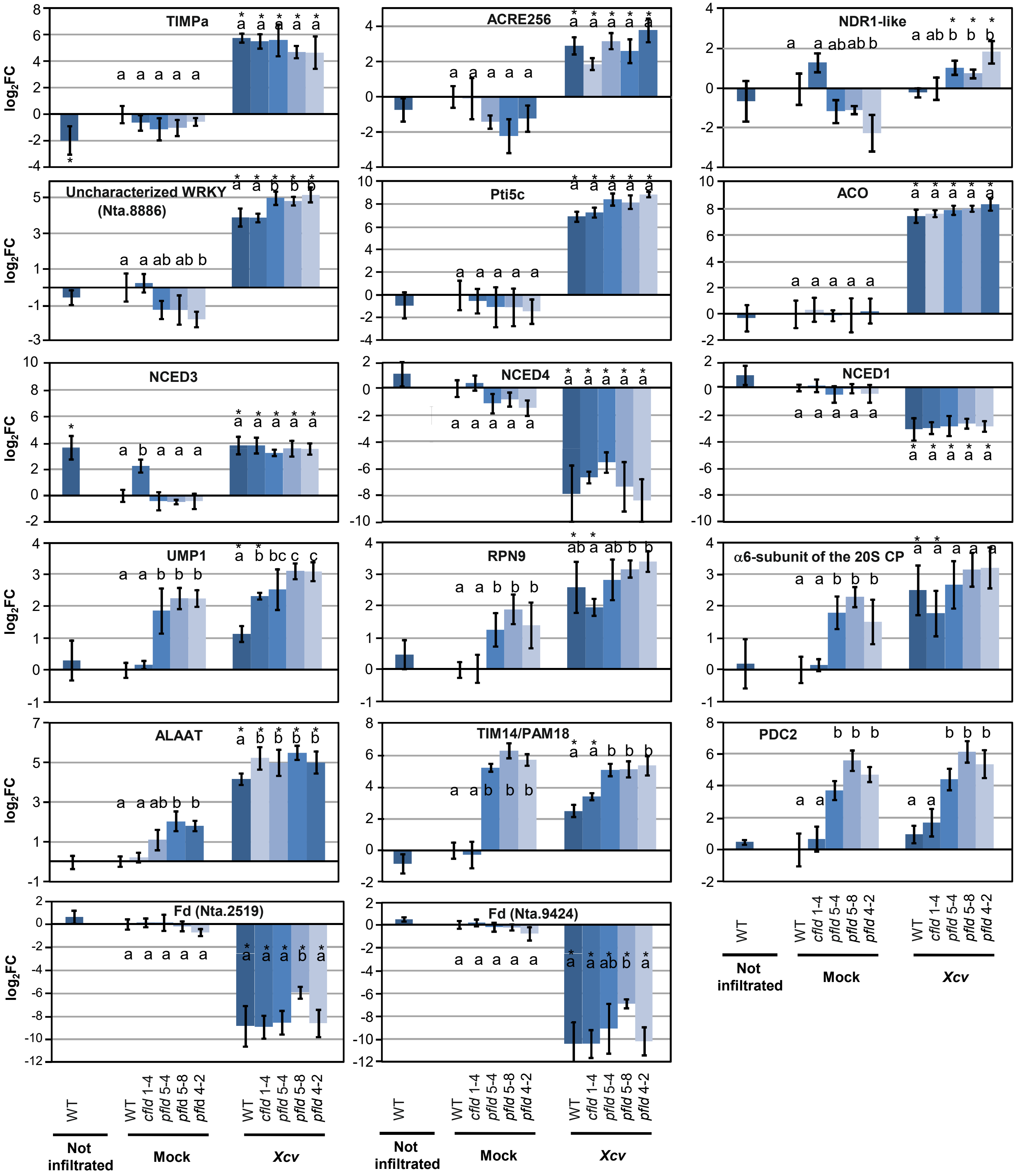

Supplementary Figure 5: Expression patterns of selected genes measured by qRT-PCR. Labelings of the abscissa corresponds to WT, *cfld*4-1, *pfld*5-4, *pfld*5-8 and *pfld*4-2 plants under mock and *Xcv* conditions. Fold-change values in the ordinates are represented in $\log_2$ scale relative to those of WT siblings infiltrated with mock solution. Each data point of qRT-PCR determinations represents the mean and standard deviation of 5-6 biological replicates and 2 technical replicates. For each condition, plant lines not sharing a common letter above the data bar are significantly different from one another (P<0.05, two-way ANNOVA). Within each line, a statistically significant effect of the infection is indicated as an asterisk.
