# Supplementary material for: Chloroplast Redox Status Modulates Genome-Wide Plant Responses during the Non-host Interaction of Tobacco with the Hemibiotrophic Bacterium Xanthomonas campestris pv. vesicatoria
Source: Front Plant Sci. 2017 Jul 4;8:1158. doi: 10.3389/fpls.2017.01158 (PMC5495832; doi:10.3389/fpls.2017.01158)
Supplement: Supplementary file 13 [file Image_6.PDF]

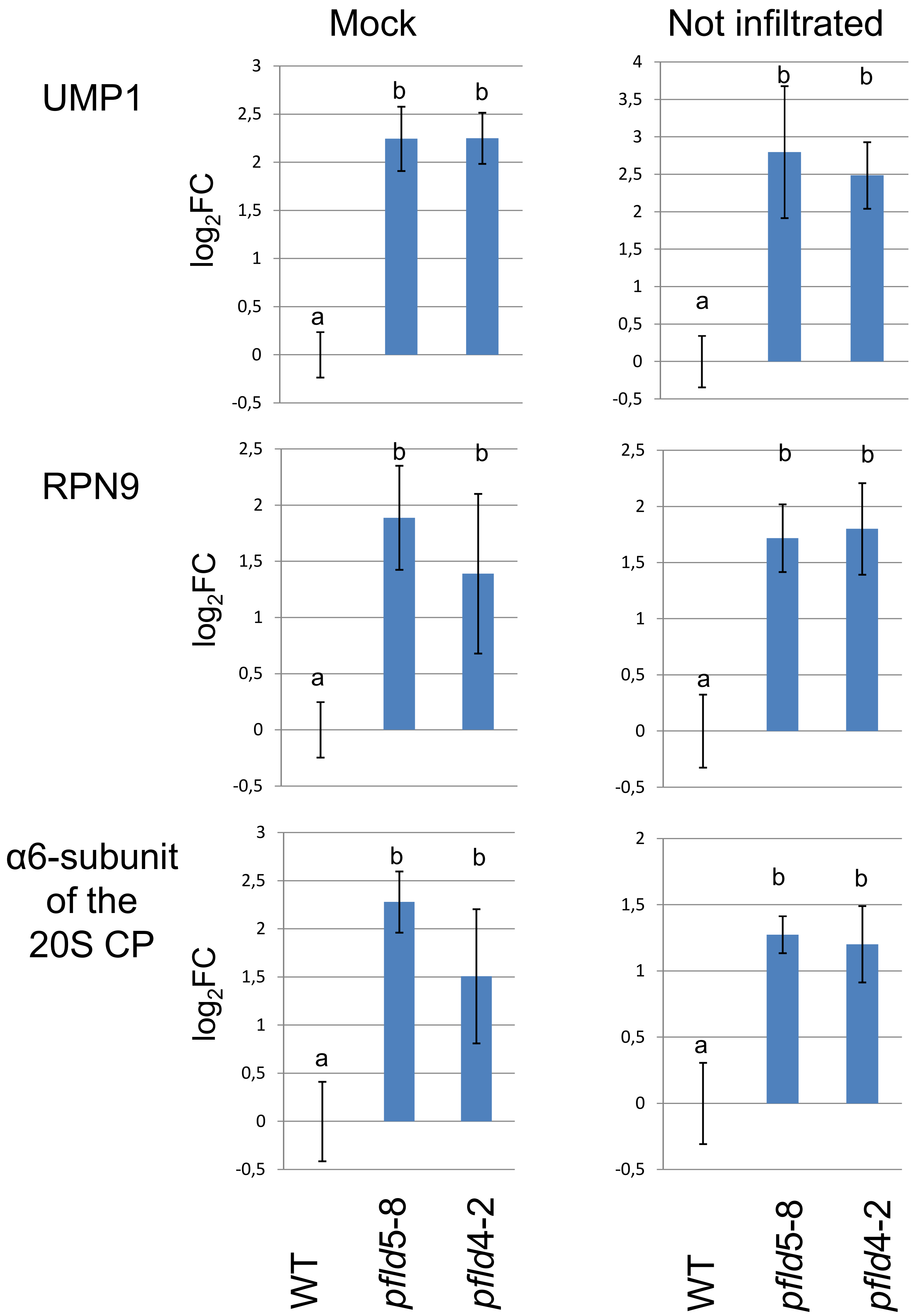

Supplementary Figure 6: Expression patterns of proteasome-coding genes measured by qRT-PCR in mock-infiltrated and non-infiltrated leaves of WT, *pfld*4-2 and *pfld*5-8 plants. Fold-change values in the ordinates are represented in $\log_2$ scale relative to those of WT siblings. Each data point of qRT-PCR determinations represents the mean and standard deviation of 4 biological replicates. Means not sharing the same letter are statistically significantly different (two Way ANNOVA, $P < 0.05$).
